# Experimental Insights into Conformational Ensembles of Assembled β-Sheet Peptides

**DOI:** 10.1021/acscentsci.3c00230

**Published:** 2023-07-04

**Authors:** Lanlan Yu, Ruonan Wang, Shucong Li, Ufuoma I. Kara, Eric C. Boerner, Boyuan Chen, Feiyi Zhang, Zhongyi Jian, Shuyuan Li, Mingwei Liu, Yang Wang, Shuli Liu, Yanlian Yang, Chen Wang, Wenbo Zhang, Yuxing Yao, Xiaoguang Wang, Chenxuan Wang

**Affiliations:** † State Key Laboratory of Common Mechanism Research for Major Diseases, Haihe Laboratory of Cell Ecosystem, Department of Biophysics and Structural Biology, Institute of Basic Medical Sciences, Chinese Academy of Medical Sciences, School of Basic Medicine Peking Union Medical College, Beijing 100005, People’s Republic of China; ‡ Department of Chemistry and Chemical Biology, Harvard University, Cambridge, Massachusetts, 02138, United States; § William G. Lowrie Department of Chemical and Biomolecular Engineering, The Ohio State University, Columbus, Ohio 43210, United States; ∥ Institute for Advanced Materials, Jiangsu University, Zhenjiang, Jiangsu 212013, People’s Republic of China; ⊥ Department of Clinical Laboratory, Peking University Civil Aviation School of Clinical Medicine, Beijing 100123, People’s Republic of China; # CAS Key Laboratory of Biological Effects of Nanomaterials and Nanosafety, CAS Key Laboratory of Standardization and Measurement for Nanotechnology, Laboratory of Theoretical and Computational Nanoscience, CAS Center for Excellence in Nanoscience, National Center for Nanoscience and Technology, Beijing 100190, People’s Republic of China; ∇ Division of Chemistry and Chemical Engineering, California Institute of Technology, Pasadena, California 91125, United States; ○ Sustainability Institute, The Ohio State University, Columbus, Ohio, 43210, United States

## Abstract

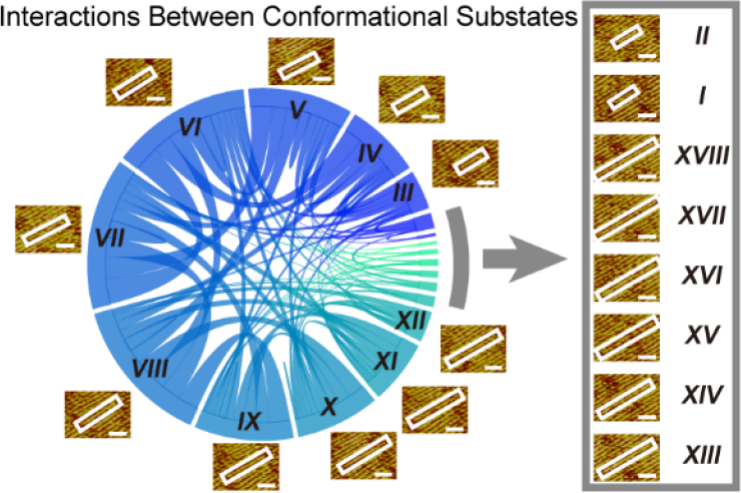

Deciphering the conformations and interactions of peptides in their assemblies offers a basis for guiding the rational design of peptide-assembled materials. Here we report the use of scanning tunneling microscopy (STM), a single-molecule imaging method with a submolecular resolution, to distinguish 18 types of coexisting conformational substates of the β-strand of the 8-37 segment of human islet amyloid polypeptide (hIAPP 8-37). We analyzed the pairwise peptide–peptide interactions in the hIAPP 8-37 assembly and found 82 interconformation interactions within a free energy difference of 3.40 *k*_B_*T*. Besides hIAPP 8-37, this STM method validates the existence of multiple conformations of other β-sheet peptide assemblies, including mutated hIAPP 8-37 and amyloid-β 42. Overall, the results reported in this work provide single-molecule experimental insights into the conformational ensemble and interpeptide interactions in the β-sheet peptide assembly.

## Abstract 2

Scanning tunneling microscopy can resolve the conformational ensembles of β-sheet peptides in a peptide homoassembly and reveal interactions between different conformational substates.

## Introduction

β-Sheet peptides are a category of basic structural elements that can be exploited as building blocks for the construction of modular protein assemblies and exquisite biomaterials for a variety of applications, including tissue engineering scaffolds, drug delivery carriers, and sensors for bioactive molecules.^1−4^ Understanding the intermolecular interactions of β-sheet peptides is critical for designing β-sheet-assembled materials.^5−13^ Conventionally, the conformation of β-sheet peptides in a single peptide assembly is thought to be uniform, though the conformations of peptides in different assemblies can be distinct.^14−16^ Accordingly, the driving forces underlying the peptide assembly have been primarily derived by assuming single and fixed interpeptide interactions. However, recent biophysical progress has indicated that a protein/peptide in solution exists as an ensemble of interchanging conformations (referred to as conformational ensembles) instead of a single, fixed conformation.^17−19^ Herein, we hypothesize that β-sheet peptides adopt multiple conformations in the assembly, which may substantially change the understanding of the intermolecular interactions underlying the β-sheet assembly. To test this hypothesis, a method that can simultaneously resolve the coexisting conformations and the interpeptide interaction among different conformations is highly demanded.

To date, cryogenic electron microscopy (cryo-EM), X-ray crystallography, and nuclear magnetic resonance (NMR) spectroscopy with dynamic experiments, including Carr–Purcell–Meiboom–Gill relaxation dispersion and chemical exchange saturation transfer, have been used to analyze protein conformational ensembles.^20−23^ These technologies have achieved high-resolution structural determination, providing insights into the three-dimensional arrangement of atoms within a protein and determining the dynamics of structural transformation between different conformational states. However, these methods require averaging distinct conformational substates, which inevitably masks the sparsely populated substates and limits the number of observable conformational states. Additionally, high-throughput structure identification is still difficult to accomplish with current technologies due to the complex and labor-intensive sample preparation (e.g., isotopic labeling for NMR and crystallization for X-ray crystallography), spectral acquisition/data collection, and structural reconstruction (e.g., single-particle reconstruction for cryo-EM).

In contrast, STM circumvents the need to average the polydisperse structures of coexisting conformational substates. STM has also been proven to be time-saving and requires no special sample preparation or data reconstruction algorithms when used in the structural characterization of organic molecule assemblies under ambient conditions.^24−26^ Herein we report the use of STM to probe the local electronic density of states of peptides, identify multiple, coexisting conformations, and elucidate the interpeptide interactions in the peptide assembly. The results obtained from STM imaging, such as the probabilistic interaction network and energetic landscape of complex interpeptide interactions within a β-sheet peptide assembly, can guide the design of peptide assemblies to achieve the desired structures and applications. We demonstrate that STM can be used as a rapid imaging method for exploring conformational structures and interpeptide interactions of assembled β-sheet peptides.

## Results and Discussion

### Formation of Homoassemblies of β-Sheet Peptides

**Figure 1:**
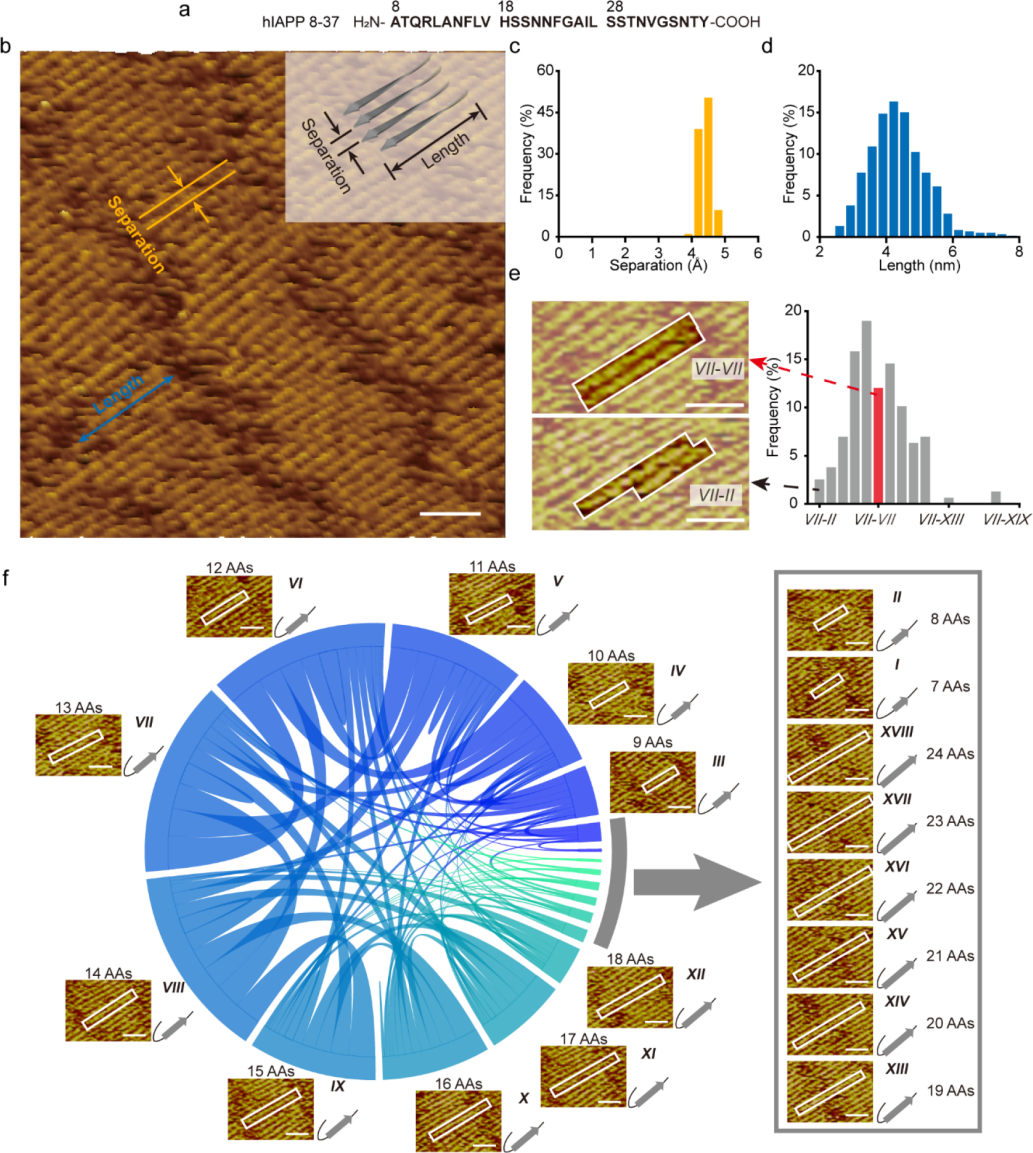
Conformational ensemble and interpeptide interactions of hIAPP 8-37. (a) The primary sequence of hIAPP 8-37. (b) A representative STM image of the hIAPP 8-37 assembly. Tunneling conditions: tunneling current 299.1 pA and bias voltage 529.8 mV. (c, d) The separation distance between two adjacent peptide β-strands and the length of peptide β-sheets. (e) STM images showing interpeptide interaction in the hIAPP 8-37 assembly and the percentage of different substates interacting with substate *VII* (*N* = 158). The interaction between the same conformational substates (*VII*–*VII*) is shown in red. (f) Conformational substates and the intersubstate interactions identified in the hIAPP 8-37 assembly. Representative STM images and length of amino acid residues in the β-strand (AA) for each conformational substate are shown with the circular chord diagram of interpeptide interactions (*N* = 554). Scale bars: 2 nm.

To test the feasibility of using STM to investigate the peptide conformations within a peptide-assembled β-sheet, we first probed and analyzed the assembled structures of the 8-37 segment of the human islet amyloid polypeptide (hIAPP 8-37; Figure 1a). The β-sheet aggregation of hIAPP is associated with the pathogenesis of type 2 diabetes.^27,28^ Previous studies have shown that hIAPP 8-37 folds into a β-hairpin and aggregates to a pleated β-sheet by forming interpeptide hydrogen bonds.^29,30^ Our Fourier-transform infrared (FTIR) spectroscopy, transmission electron microscopy (TEM), and circular dichroism (CD) spectroscopy experiments confirm the formation of β-sheets by hIAPP 8-37, which was equilibrated at 37 °C for 48 h (Figures S1 and S2).

### STM Imaging and Conformational Analysis of β-Sheet Peptides in Assemblies

By measuring the hexagonal atomic carbon images on the highly oriented pyrolytic graphite (HOPG) surface, the STM spatial resolution can be determined to be higher than 0.5 Å in the vertical direction and higher than 2.6 Å in the horizontal direction (Figure S3). The equilibrated hIAPP 8-37 assemblies on a freshly cleaved HOPG surface display lamellar-like structures in the STM image (Figure 1b). Single-molecule imaging of ∼1000 individual peptide molecules was completed within 2 min with a scan range of 100 nm × 100 nm (512 pixels × 512 pixels) and a scan rate of 4–5 Hz. The bright stripes correspond to the β-strand regions of hIAPP 8-37. The average separation between two neighboring peptide strands is 4.4 ± 0.2 Å (Figure 1c), consistent with the interstrand spacing in a β-sheet peptide assembly.^27^ The STM image exhibits a variable β-strand length of hIAPP 8-37 molecules to form pleated peptide-assembled β-sheets (Figure 1b,d). Since the separation between two neighboring residues within one β-strand is 3.25 Å, the number of amino acid residues of the β-strand can be deduced from the length of the β-strand.^31^ Based on the residue number of β-strands, the conformational ensemble of hIAPP 8-37 β-strand includes 18 types of distinct conformational substates labeled from *I* to *XVIII* (Figures S4–S6), and the most probable substates are *VI* (14.85%), *VII* (16.34%), and *VIII* (15.02%). It is worth noting that the invisible portion of peptides in the STM images is due to their distance from the surface exceeding the tunneling distance between the STM probe and the substrate (∼1 nm). Only the portion of the adsorbed peptides that is within the tunneling distance to the HOPG surface, enabling the tunneling electrons to penetrate across the energy gap between the STM probe and substrate, is visible in the STM images.

### Study of the Interactions between Conformational Substates Using STM

Next, we sought to analyze the interpeptide interactions between different hIAPP 8-37 conformational substates. Within a β-sheet peptide assembly, each β-strand is flanked by two adjacent β-strands, forming two categories of interpeptide interactions: (1) homointeractions, e.g., *VII*–*VII* interactions, and (2) heterointeractions, e.g., *II–VII* interactions. Before describing the results regarding interpeptide interactions, we must state that it is difficult to distinguish different fine structures of a single peptide when the length of a β-strand is fixed based on the current resolution. Take substate *VII* as an example (Figure 1e); it interacts with the other 12 types of substates (from *II* to *XVII*), and the most probable type of interpeptide interaction is *VI*–*VII*, which accounts for 19.0% of the total interpeptide interactions. As summarized in the circular chord diagram of interpeptide interactions identified in the hIAPP 8-37 assemblies (Figure 1f), the arc length of the sector reflects the population proportion of the corresponding conformational substate. The thickness of the curves connecting different substates indicates the probability of the corresponding interaction, with a thicker curve representing a higher probability of such an interaction within the distribution of interstrand interactions.

### STM Unveils Peptide Conformational Ensemble Changes Caused by Single-Site Substitutions

**Figure 2:**
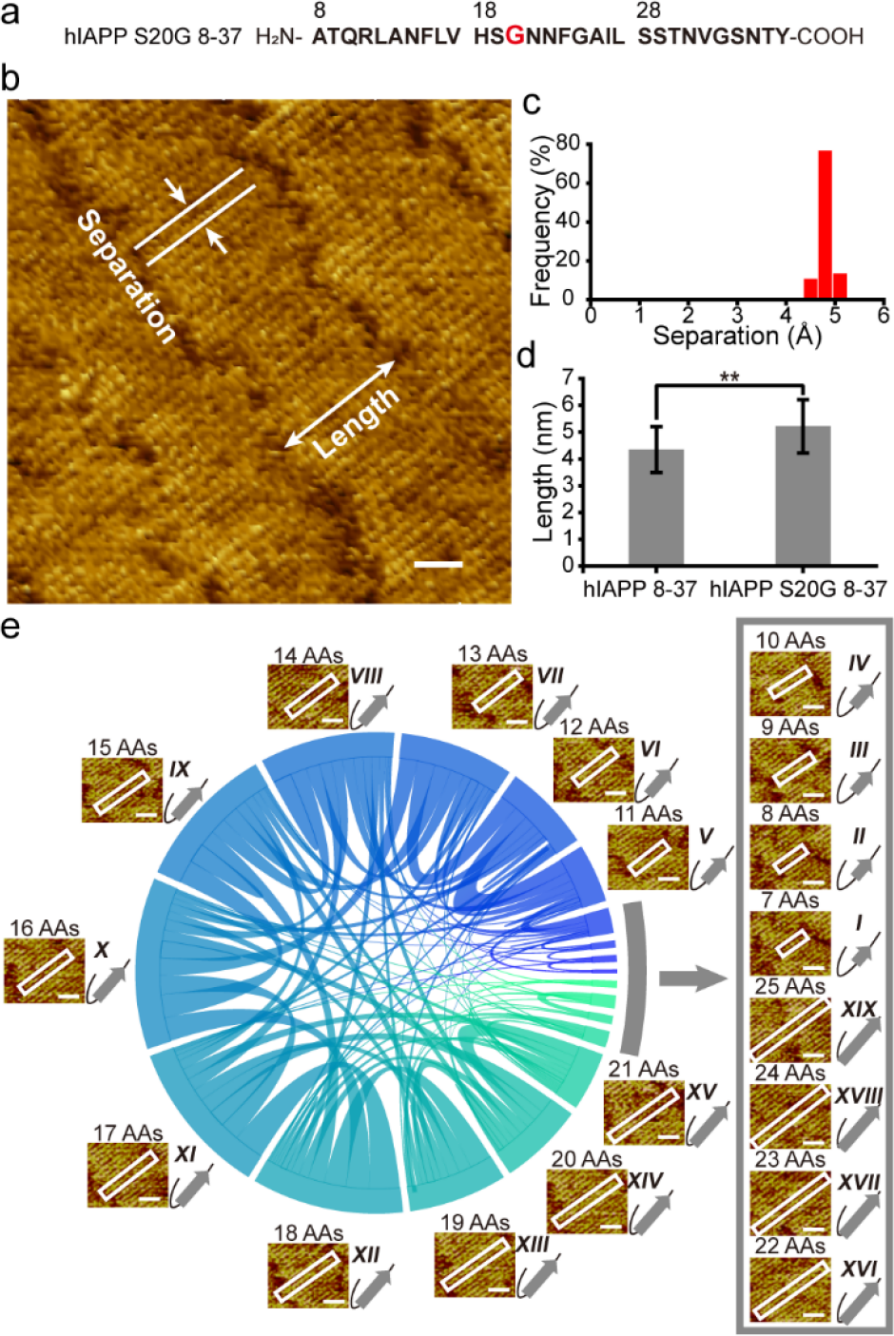
Conformation ensemble and interpeptide interactions of hIAPP S20G 8-37. (a) The primary sequence of hIAPP S20G 8-37. (b) A representative STM image of the hIAPP S20G 8-37 assembly. Tunneling conditions: tunneling current 299.1 pA and bias voltage 600.0 mV. (c) The separation distance between two adjacent hIAPP S20G 8-37 strands. (d) A comparison of the β-strand lengths of hIAPP 8-37 and hIAPP S20G 8-37. (e) Conformational substates and the intersubstate interactions identified in the hIAPP S20G 8-37 assembly. Representative STM images and length of amino acid residues in the β-strand (AA) for each conformational substate are shown with the circular chord diagram of interpeptide interactions (*N* = 477). Scale bars: 2 nm.

The above results demonstrate that STM can identify coexisting conformational ensembles in a β-sheet peptide assembly. Next, we sought to study the effect of a single-site mutation in the peptide sequence on the peptide conformational ensemble. We selected the 8-37 segment of the hIAPP S20G (hIAPP S20G 8-37) assembly, a mutant of hIAPP with a substitution of Ser-to-Gly at site 20, as shown in Figure 2a. Previous studies have demonstrated that the S20G substitution in the hIAPP gene, found in East Asian populations, is linked to an earlier onset of type 2 diabetes compared to individuals without this substitution.^32−34^ The hIAPP S20G variant exhibits a greater propensity for β-sheet aggregation and increased cytotoxicity compared to the wild-type hIAPP.^32−34^ As evidenced in Figure 2b–d and Figures S7 and S8, hIAPP S20G 8-37 adopts a β-sheet conformation, as the lamellar-like structures, and the substitution of S20G increases the average length distribution of β-strands from 4.3 ± 0.9 nm (hIAPP 8-37) to 5.2 ± 1.0 nm (hIAPP S20G 8-37). An ensemble of 19 types of conformational substates has been identified in the hIAPP S20G 8-37 assemblies that range from 7 to 25 amino acid residues in the β-motifs (Figure 2e and Figures S9–S11). In addition, 95 interpeptide interactions among 19 coexisting substates have been identified (Figure 2e).

**Figure 3:**
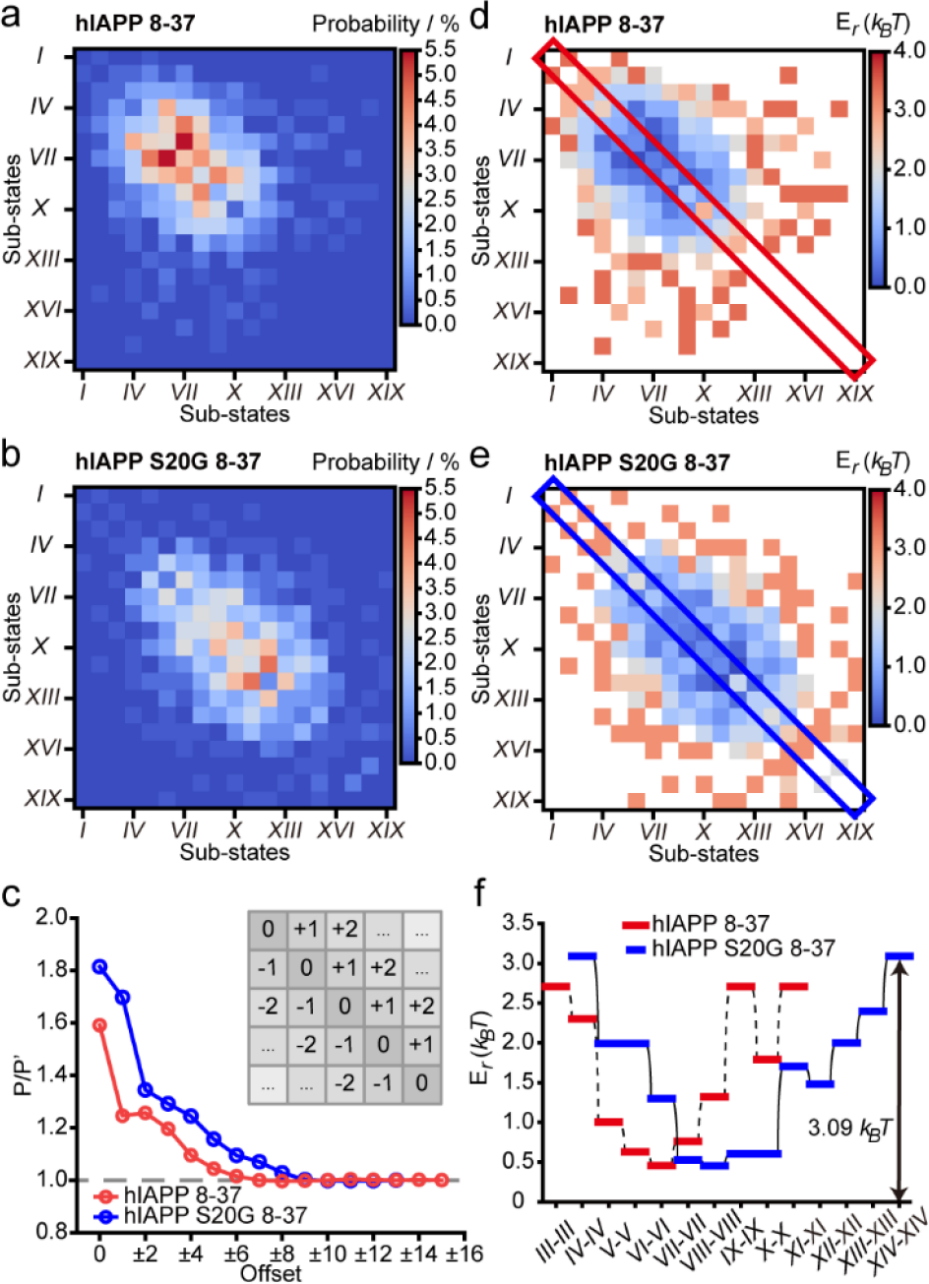
Single-site mutation modulates hIAPP 8-37 interpeptide interactions. (a, b) The probability of intersubstate interactions in the assembly of hIAPP 8-37 (*N* = 554) (a) and hIAPP S20G 8-37 (*N* = 477) (b). (c) *P*/*P′* as a function of offset for hIAPP 8-37 (red) and hIAPP S20G 8-37 (blue). The inset is a scheme showing the offset in interactions between two substates. (d, e) Two-dimensional energy landscape of the hIAPP 8-37 (d) and hIAPP S20G 8-37 (e) interpeptide interactions. (f) A comparison of free energy of homoconformational interactions (diagonal of (d) and (e)) in hIAPP 8-37 and hIAPP S20G 8-37 assemblies.

To reveal the influence of the single-site mutation on the interpeptide interactions, the two-dimensional probability matrices of interconformational interactions of hIAPP 8-37 and hIAPP S20G 8-37 assemblies are compared in Figure 3a,b. For hIAPP 8-37, the interactions between *V*–*VII* and *VI–VII* substates have the highest proportions (4.51% and 5.42%) for the interactions between the same and different conformational substates, respectively. In contrast, for hIAPP S20G 8-37, the most populated interactions are *XI*–*XII*, *IX*–*X*, and *X*–*XII*, accounting for 4.61%, 3.56%, and 3.56% of the total population of interpeptide interactions, respectively (Figure 3b). To analyze the behavior of hIAPP 8-37 and hIAPP S20G 8-37, we employed a two-dimensional probability matrix based on a random probability distribution model, specifically the Fisher–Yates shuffle algorithm. In this model, the molecules are treated as ideal gases, assumed to move freely without mutual interactions, resembling a noninteracting system. Therefore, the dominant factor governing the interpeptide interactions within this model is entropy (Figures S12 and S13). We calculated the sum of the interaction probability along the matrix diagonal (with a specific offset) of experimental measurements (*P*) and the calculated random permutation (*P*′) (Figure 3c and Figure S14). The offset represents the relative length of the β-sheet in the neighboring peptide. e.g., offset = +1/–1 represents that the substate of the neighboring peptide has one more/less residue in its β-sheet. An inspection of *P*/*P*′ values in Figure 3c leads us to draw two conclusions. First, *P*/*P*′ > 1 for offsets ranging from 0 to ±3 reveals that enthalpic interaction favors the interaction between substates with similar β-strand lengths and overcomes the entropic contribution. Second, the pathological single-site mutation S20G enhances the enthalpic interaction between substates with similar β-strand length (1.70 for hIAPP S20G 8-37 and 1.25 for hIAPP 8-37 at an offset of ±1). This observation suggests that the S20G substitution facilitates the interpeptide interactions for the assembly of hIAPP S20G 8-37 β-sheets, which is consistent with past studies showing a high tendency of hIAPP S20G in β-sheet aggregation.^34^

We further calculated the relative free energies of each type of interconformational interaction. For a peptide-assembled system at equilibrium, the relative free energy difference (*E*_*i*_ – *E*_*j*_) between two types of interconformation interactions, *i* and *j*, can be related to their probabilities (*P*_*i*_ and *P*_*j*_) by the Boltzmann distribution^35^
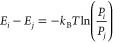
1where *k*_B_ is the Boltzmann constant and *T* is the absolute temperature of the system. We used the most probable types of interpeptide interactions as the reference states (*VI–VII* for hIAPP 8-37 and *XI*–*XII* for hIAPP S20G 8-37). A comparison of the two-dimensional energetic landscape of the two peptides (Figure 3d,e) reveals that the single-site mutation moves the most probable interpeptide interactions toward a longer β-strand (the diagonal toward the bottom right corner). The free energies of homoconformational interactions in hIAPP 8-37 and hIAPP S20G 8-37 assemblies are compared in Figure 3f. These results reveal the significant effect of the single-site mutation on the conformational ensemble and interpeptide interactions. Furthermore, through STM experiments, we can analyze the impacts of the S20G substitution on the observed variations in single β-sheet assemblies. By comparing STM images and analyzing β-strand length distributions and interconformational interactions, we gain valuable insights into how the S20G substitution influences the structural characteristics and dynamics of individual peptide assemblies. This comprehensive analysis enables us to investigate the specific effects of the S20G substitution on the formation and stability of β-sheet assemblies at the single-molecule level (Figures S15 and S16).

### Generality of STM-Based Conformational Analysis of β-Sheet Peptides

**Figure 4:**
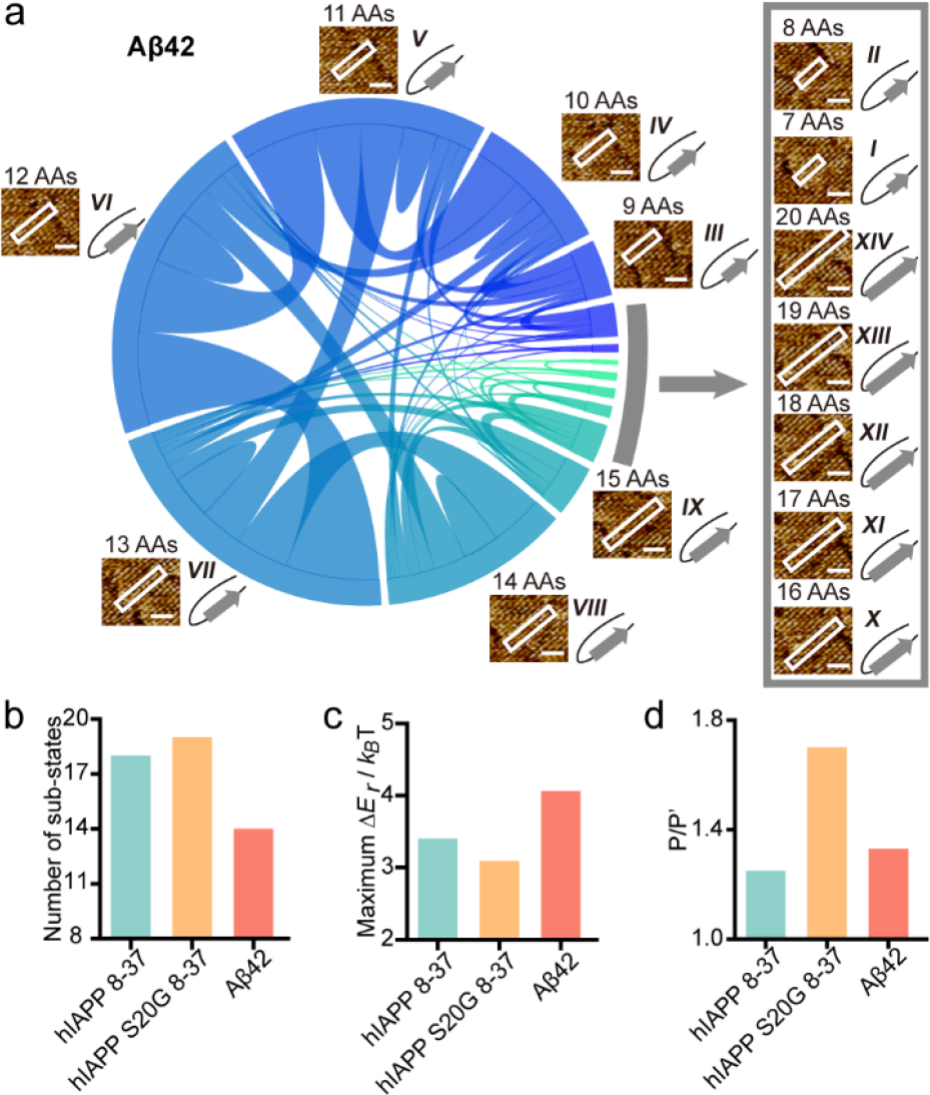
Conformation ensemble and interpeptide interactions of Aβ42. (a) Conformational ensemble and interpeptide interactions of Aβ42 (*N* = 460). Representative STM images and lengths of amino acid residues in the β-strand (AA) for each conformational substate are shown with the circular chord diagram of interpeptide interactions. Scale bars: 2 nm. (b–d) A comparison of the number of conformational substates (b), the energy difference of the conformational ensemble (c), and *P*/*P*′ at an offset of ±1 (d) of hIAPP 8-37, hIAPP S20G 8-37, and Aβ42.

Finally, to test the generality of our STM-based method, we used STM to image the assembly of another β-sheet peptide, amyloid-β 42 (Aβ42) (Figure 4a and Figures S17 and S18). Aβ42 assembly forms lamella structures with an average inter-β-strand separation of 4.2 ± 0.1 Å, as evidenced in Figure S19. Specifically, 14 conformational substates and 53 types of interpeptide interactions were identified in the Aβ42 assembly (Figure 4a and Figures S20–S24). The analysis of the energetic landscapes of the three β-sheet peptides reported in this work leads us to make three main conclusions. First, the conformations of assembled peptides within a peptide-assembled β-sheet are unsynchronized and heterogeneous. The number of concurrent substates as identified for each peptide assembly fluctuates from 14 (Aβ42) to 19 (hIAPP S20G 8-37), highlighting the complexity of the intermolecular interactions that harness the assembly of a β-sheet (Figure 4b). Second, distinct interpeptide interactions within a peptide-assembled β-sheet are observed to be close in free energy, where the maximum energy differences in the energetic landscapes of interpeptide interactions fluctuate within 4.10 *k*_B_*T* for the three types of peptides (Figure 4c). The proximity of interconformation interactions in energy leads to the finding that there does not exist a predominant interpeptide interaction that determines the supramolecular organization of assembled peptides. Third, β-sheet peptides favor the interaction between substates with similar β-strand lengths (e.g., *P*/*P*′ > 1 for all three β-sheet peptides at an offset of ±1) (Figure 4d).

## Conclusion

In conclusion, we demonstrate that single-molecule imaging based on STM can effectively resolve the structures of conformational substate ensembles and reveal the interpeptide interactions in β-sheet peptides. Our imaging results reveal the chaotic nature of the self-assembly of β-sheet peptides, where many metastable conformational substates coexist and recognize each other. Compared with other techniques, such as cryo-EM and NMR, the STM experiment offers several advantages, including a relatively simple and rapid sample preparation, imaging, and data analysis process. Additionally, STM exhibits high sensitivity to lowly populated conformational substates, as it avoids the averaging of heterogeneous structures that can occur in other techniques. The peptide-assembled structures can be constructed by engineering an ensemble of heterogeneous interpeptide interaction modes to go beyond the simple interactions encoded by the lowest free energy conformation. This approach can benefit studies aiming to understand the impact of structural variation, such as amino acid mutation and post-translational modification, on the pathologic mechanism of protein misfolding diseases, including prion diseases, Parkinson’s disease, amyloidosis, etc.

## Materials and Methods

### Reconstitution of Peptide Assemblies in Solution

The peptides were synthesized and purified by Bankpeptide Biological Technology Co., Ltd., ensuring a high quality with a purity above 98% as verified by high-performance liquid chromatography and mass spectroscopy. To unfold the peptides, 1,1,1,3,3,3-Hexafluoro-2-propanol (HFIP, Innochem) was utilized and subsequently evaporated by using nitrogen gas. The peptides were then refolded and assembled by redissolving them in Milli-Q water at concentrations of 40 μM for hIAPP 8-37, 40 μM for hIAPP S20G 8-37, and 40 μM for Aβ42. The peptide assemblies were equilibrated in solution at 37 °C for 48 h.

The sequences of β-sheet peptides are as follows.

hIAPP 8-37: H_2_N-ATQRLANFLV HSSNNFGAIL SSTNVGSTNY-COOH

hIAPP S20G 8-37: H_2_N-ATQRLANFLV HSGNNFGAIL SSTNVGSTNY-COOH

Aβ42: H_2_N-DAEFRHDSGY EVHHQKLVFF AEDVGSNKGA IIGLMVGGVV IA-COOH

### Peptide Concentration Determination

To unfold the peptides, we dissolved them in a 6 M guanidinium chloride aqueous solution. The concentrations of the peptides were determined using a UV–vis spectrophotometer (PerkinElmer, USA). The UV absorbance (*A*) of the peptides at a wavelength of 280 nm was correlated with the molar extinction coefficient of the peptides (ε), the optical path length of the cell (*L*), and the peptide concentration (*c*) according to the Beer–Lambert law equation:
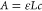
2

### Single-Molecule Imaging by STM

A 10 μL aliquot of the peptide solution was deposited onto the freshly cleaved surface of HOPG and allowed to dry. Under ambient conditions, single-molecule imaging experiments were conducted using a Nanoscope IIIa scanning probe microscope (SPM) system in constant-current mode (Bruker, USA). STM tips were fabricated from a Pt/Ir wire (80/20). The STM experiments were independently repeated using different samples and tips.

### Interpeptide Interaction Analysis

The lengths of the peptide strands (*N* > 500) in the STM images were measured using Nanoscope software (Bruker, USA). Assignments of the conformational substates were determined from the β-strand lengths. The neighboring peptide conformations for each conformational substate were identified from the STM images and counted. In principle, the determination and statistical analysis of interpeptide interactions can be completed by either manual measurement or automated analysis using image software.

### FTIR Spectroscopy Measurements

A 20 μL aliquot of the peptide solution was deposited on a calcium fluoride window. Once the peptide solutions were dried, the FTIR spectra were recorded by using a PerkinElmer Spectrum One FTIR Spectrometer (Waltham, Massachusetts, USA). The spectral range covered wavenumbers from 4000 to 4500 cm^–1^ with an increment of 2 cm^–1^. PeakFit software (Version 4.12, San Jose, CA, USA) and Origin software (Version 9.1, Northampton, MA, USA) were utilized to deconvolute and analyze the second-order derivative of the FTIR spectra.

### TEM Measurements

To observe the peptide assemblies, 10 μL of the peptide solution was deposited onto a 200 mesh Formvar carbon-coated copper TEM grid and allowed to settle for 2 min at room temperature. Excess solution was removed using filter paper, and the samples were dried for 2 h at room temperature. Staining was performed using 1% phosphotungstic acid for 30 s. After being rinsed with Milli-Q water three times, the grids were thoroughly dried before TEM characterization. A Hitachi H-7650 transmission electron microscope (Hitachi, Tokyo, Japan) was used to visualize the topological features of the peptide assemblies.

### CD Analysis

CD spectra of the peptide solutions were recorded at room temperature by using a circular dichroism spectropolarimeter system (Jasco J-1500, Japan). The CD cuvette had a path length of 0.1 mm. The wavelength range covered from 190 to 320 nm with a bandwidth of 2 nm. The scan speed was set to 50 nm/min, and the digital integration time was 1 s. The CD signal obtained from the solvent alone was used as a background reference and subtracted from the CD signal collected from the peptide samples.

## Supporting Information Available

FTIR spectroscopy, CD spectroscopy, and TEM images of peptide assemblies, details of STM experiments and analyses, including length distribution of the peptide β-strands, population distribution of peptide conformational substates, the probability of interconformation interactions calculated for hIAPP 8-37 and hIAPP S20G 8-37 based on a random distribution model, number distribution of different types of interconformation interactions identified from the STM experiments for Aβ42, and energy landscape of the interpeptide interactions identified from the STM experiments for Aβ42 (PDF)
