# Supplementary material for: Experimental Insights into Conformational Ensembles of Assembled β-Sheet Peptides
Source: ACS Cent Sci. 2023 Jul 4;9(7):1480–7. doi: 10.1021/acscentsci.3c00230 (PMC10375872; doi:10.1021/acscentsci.3c00230)
Supplement: Supplementary file 1 — oc3c00230_si_001.pdf [file oc3c00230_si_001.pdf]

Supporting Information

Experimental Insights into Conformational Ensembles of Assembled β-Sheet Peptides

Lanlan Yu[1,‡], Ruonan Wang[1,‡], Shucong Li[2], Ufuoma I. Kara[3], Eric C. Boerner[3], Boyuan Chen[3], Feiyi Zhang[1,4], Zhongyi Jian[1], Shuyuan Li[1], Mingwei Liu[1], Yang Wang[1], Shuli Liu[5], Yanlian Yang[6], Chen Wang[6], Wenbo Zhang[1,], Yuxing Yao[7], Xiaoguang Wang[3,8,*], and Chenxuan Wang[1,*]*

[1] State Key Laboratory of Common Mechanism Research for Major Diseases, Haihe Laboratory of Cell Ecosystem, Department of Biophysics and Structural Biology, Institute of Basic Medical Sciences Chinese Academy of Medical Sciences, School of Basic Medicine Peking Union Medical College, Beijing 100005, P. R. China

[2] Department of Chemistry and Chemical Biology, Harvard University, Cambridge, MA, 02138, USA

[3] William G. Lowrie Department of Chemical and Biomolecular Engineering, The Ohio State University, Columbus, OH 43210, USA

[4] Institute for Advanced Materials, Jiangsu University, Zhenjiang, Jiangsu 212013, P. R. China

[5] Department of Clinical Laboratory, Peking University Civil Aviation School of Clinical Medicine, Beijing 100123, China

[6] CAS Key Laboratory of Biological Effects of Nanomaterials and Nanosafety, CAS Key Laboratory of Standardization and Measurement for Nanotechnology, Laboratory of

Theoretical and Computational Nanoscience, CAS Center for Excellence in Nanoscience, National Center for Nanoscience and Technology, Beijing 100190, P. R. China

[7] *Division of Chemistry and Chemical Engineering, California Institute of Technology, Pasadena, CA, 91125, USA*

[8] *Sustainability Institute, The Ohio State University, Columbus, OH, 43210, USA*

[‡] L.Y. and R.W. contributed equally to this work.

[*] Corresponding authors.

Prof. Chenxuan Wang, email: wangcx@ibms.pumc.edu.cn;

Prof. Xiaoguang Wang, email: wang.12206@osu.edu;

Prof. Wenbo Zhang, email: zwb@ibms.pumc.edu.cn.

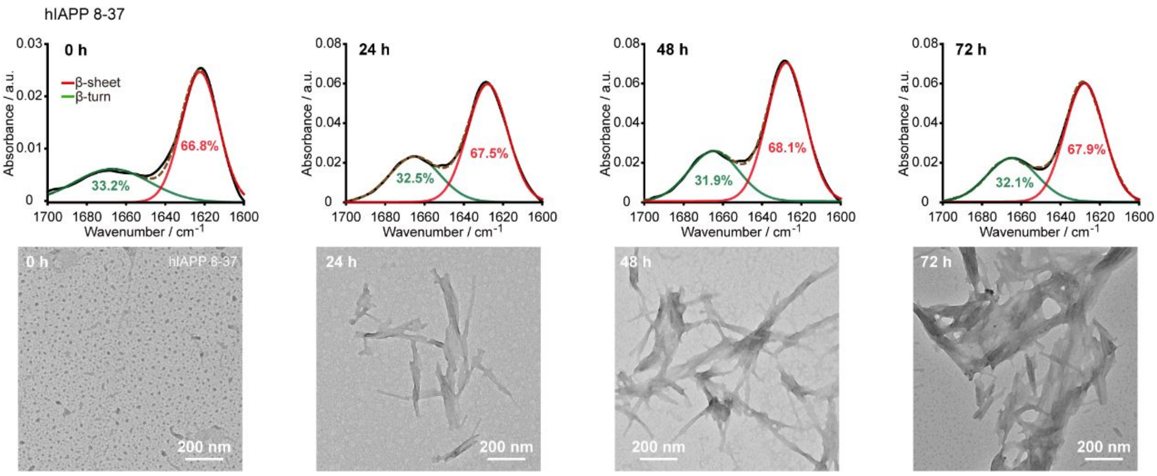

Fig. S1 hIAPP 8-37 β-sheet assembly. The second derivative FTIR spectra and TEM images of hIAPP 8-37 peptide at different equilibration times. FTIR: black solid line, experimental curve; brown dashed line, fitting curve; red peaks, β-sheet component; green peaks, β-turn component.

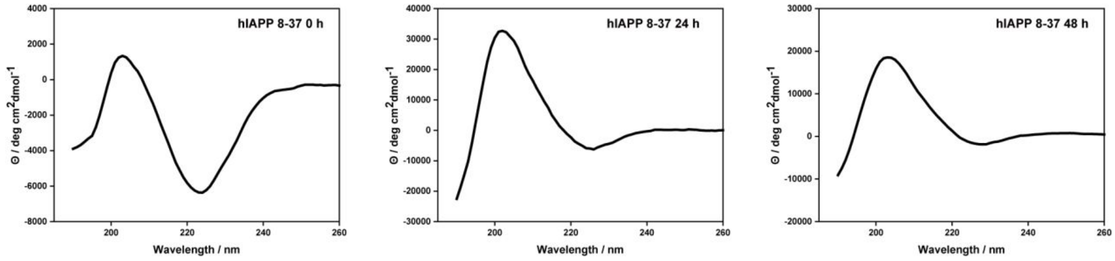

Fig. S2 CD spectra of hIAPP 8-37 at various equilibration times.

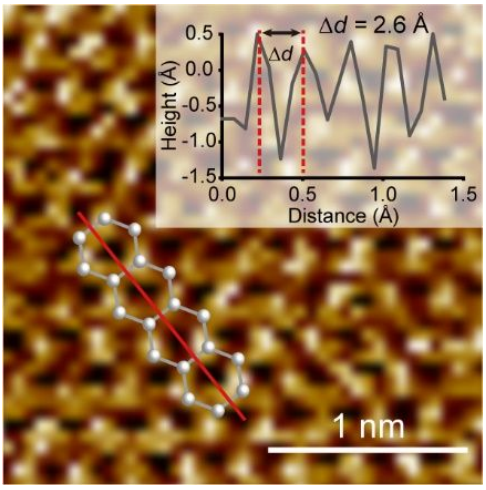

Fig. S3 STM image of an HOPG surface. A representative STM image of an HOPG surface. Tunneling condition: tunneling current is 600.0 pA and bias voltage is 10.0 mV. The cross-sectional profile corresponds to the red line in the STM image.

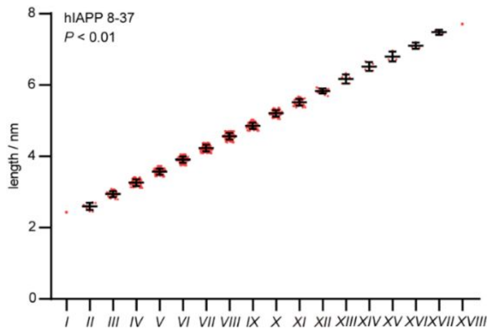

Fig. S4 Length distribution of the hIAPP 8-37 β-strands determined from the STM images in Fig. 1 of the main text.

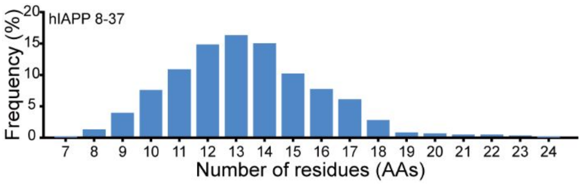

Fig. S5 Population distribution of hIAPP 8-37 β-strands in different amino acid residue number ($N = 606$).

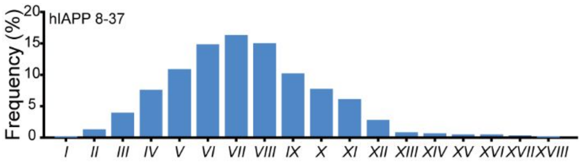

Fig. S6 Population distribution of hIAPP 8-37 conformational sub-states ($N = 606$).

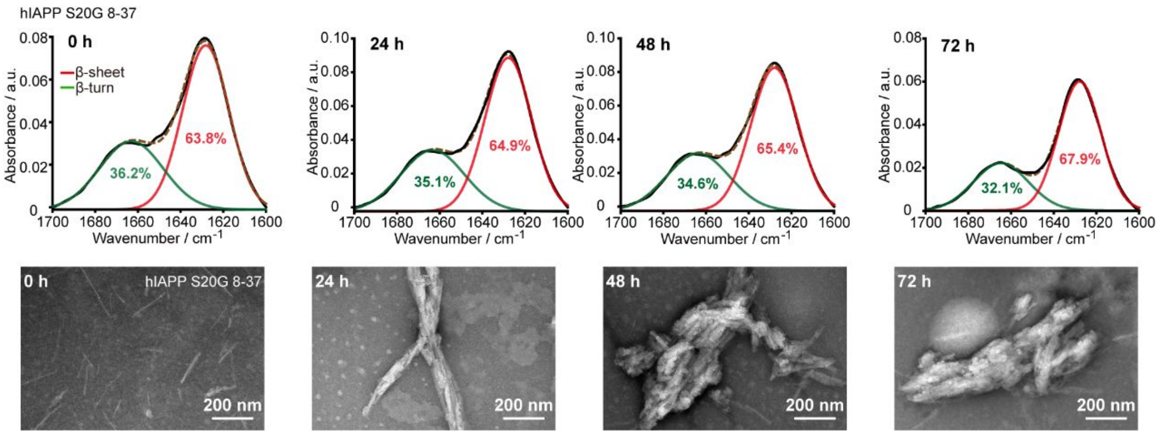

Fig. S7 hIAPP S20G 8-37 β-sheet assembly. The second derivative FTIR spectra and TEM images of hIAPP S20G 8-37 peptide at different equilibration times. FTIR: black solid line, experimental curve; brown dashed line, fitting curve; red peaks, β-sheet component; green peaks, β-turn component.

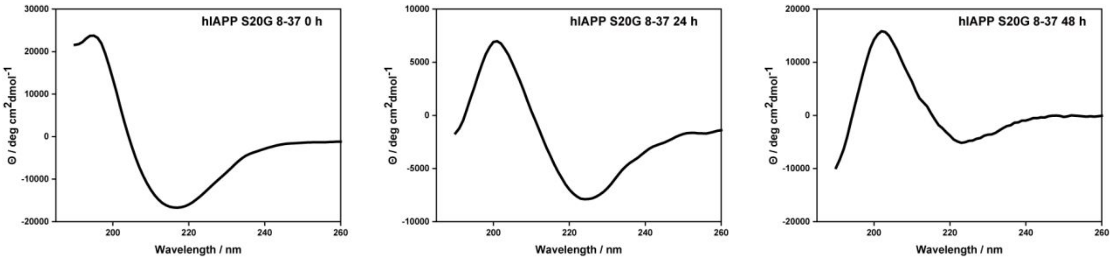

Fig. S8 CD spectra of hIAPP S20G 8-37 at various equilibration times.

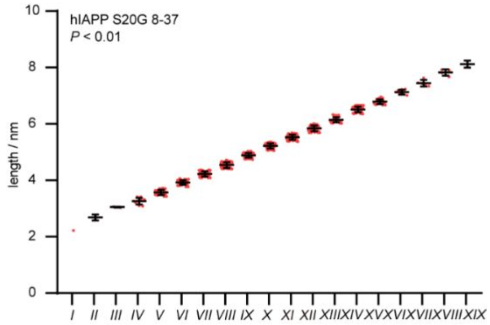

Fig. S9 Length distribution of the hIAPP S20G 8-37 β-strands determined from the STM images in Fig. 2 of the main text.

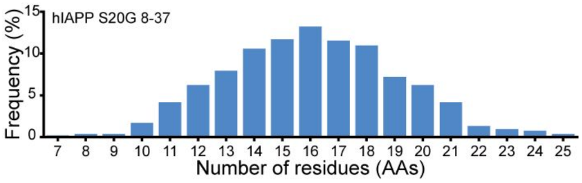

Fig. S10 Population distribution of hIAPP S20G 8-37 β-strands in different amino acid residue number ($N = 529$).

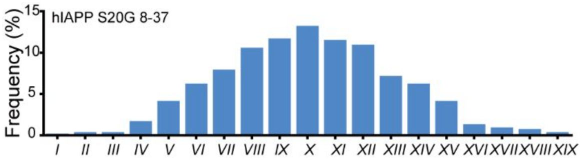

Fig. S11 Population distribution of hIAPP S20G 8-37 conformational sub-states ($N = 529$).

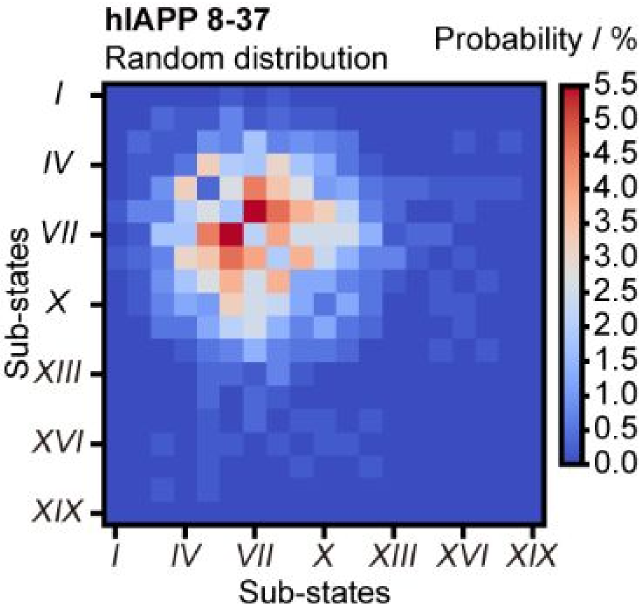

Fig. S12 The probability of inter-conformation interactions calculated for hIAPP 8-37 based on a random distribution model.

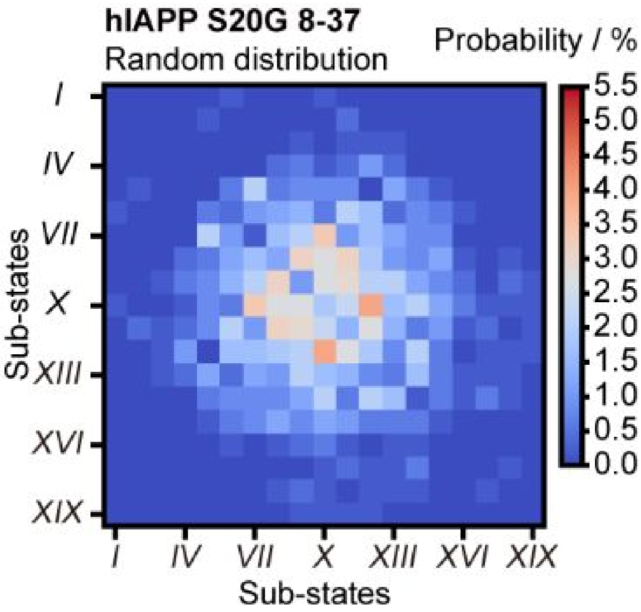

Fig. S13 The probability of inter-conformation interactions calculated for hIAPP S20G 8-37 based on a random distribution model.

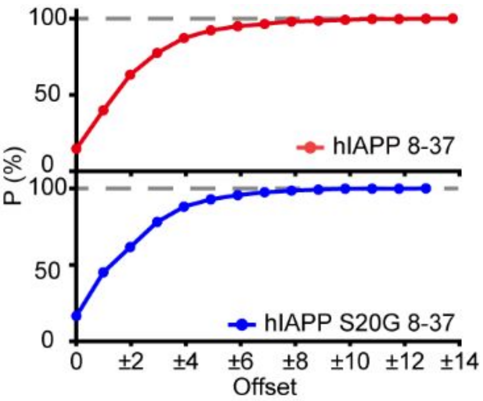

Fig. S14 The plot of *P* against offset for the hIAPP 8-37 (red) and hIAPP S20G 8-37 (blue) inter-conformation interactions determined from Fig. 3a and Fig. 3b of the main text, respectively.

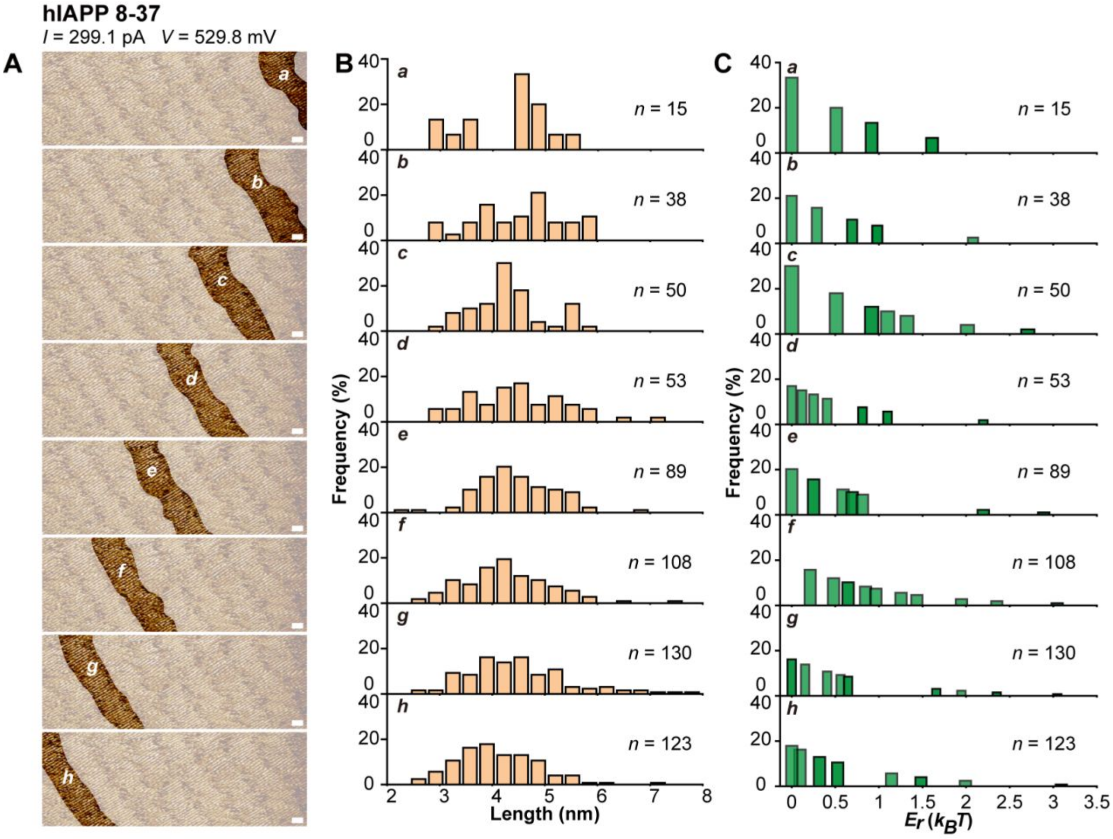

Fig. S15 (A) STM images of individual hIAPP 8-37 self-assembled β-sheets, labeled from *a* to *h*, on an HOPG surface. Scale bars, 2 nm. (B) Distribution of peptide β-strand lengths within an individual β-sheet. (C) Distribution of interactions between conformational sub-states within an individual β-sheet.

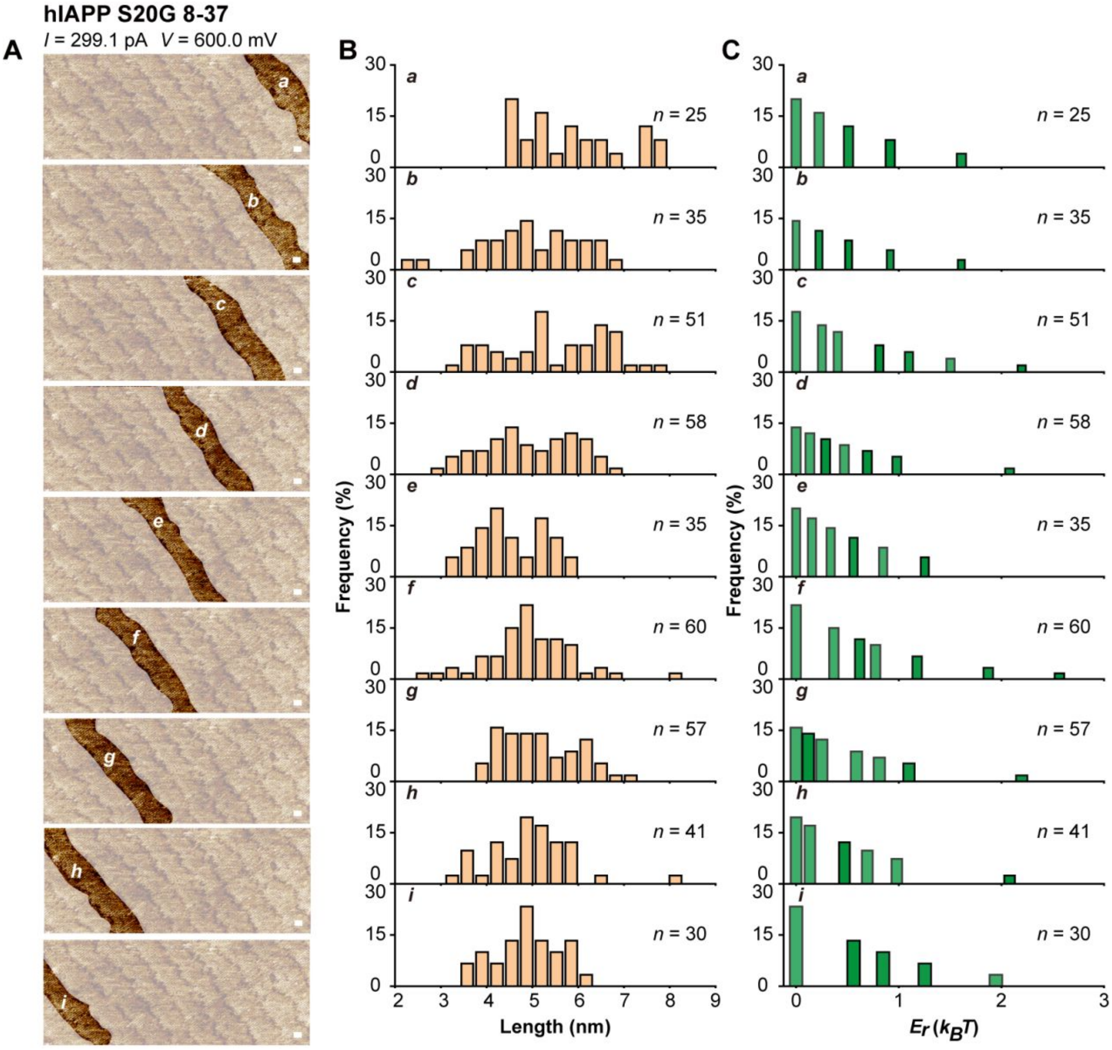

Fig. S16 (A) STM images of individual hIAPP S20G 8-37 self-assembled β-sheets, labeled from *a* to *i*, on an HOPG surface. Scale bars, 2 nm. (B) Distribution of peptide β-strand lengths within an individual β-sheet. (C) Distribution of interactions between conformational sub-states within an individual β-sheet.

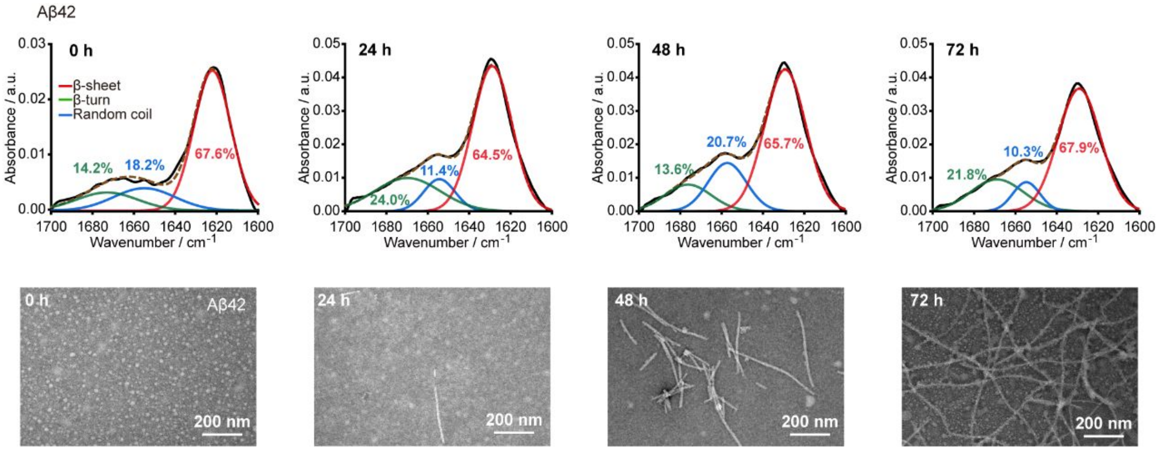

Fig. S17 Aβ42 β-sheet assembly. The second derivative FTIR spectra and TEM images of Aβ42 peptide at different equilibration times. FTIR: black solid line, experimental curve; brown dashed line, fitting curve; red peaks, β-sheet component; green peaks, β-turn component; blue peaks, random coil component.

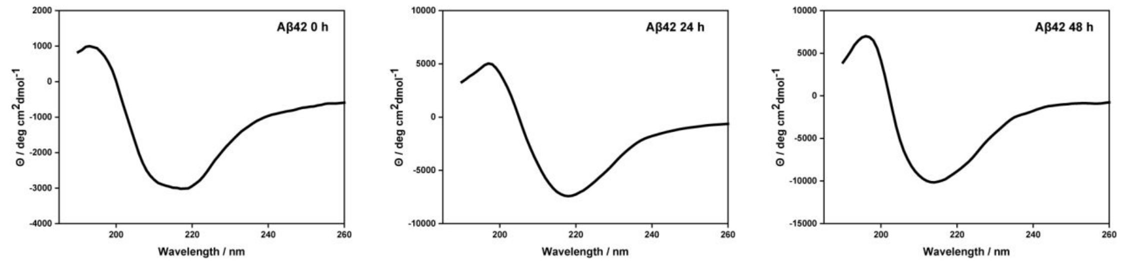

Fig. S18 CD spectra of Aβ42 at various equilibration times.

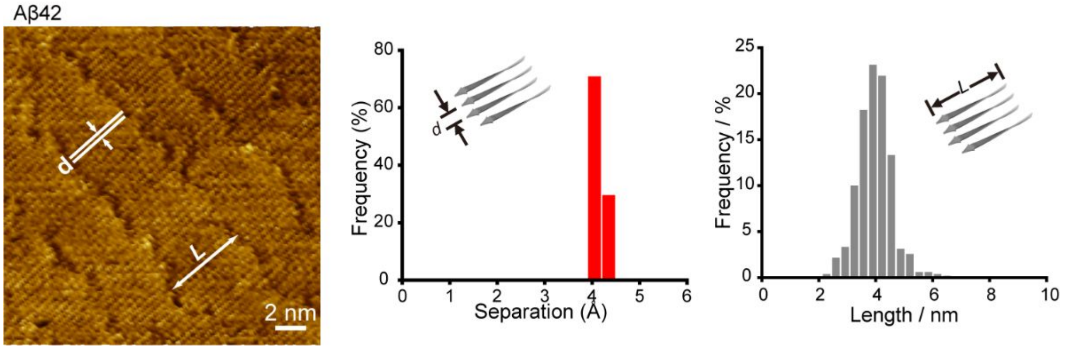

Fig. S19 Aβ42 assembly on an HOPG surface. Representative STM images of the Aβ42 self-assembly on a HOPG surface. Tunneling condition: tunneling current is 299.1 pA and bias voltage is 699.8 mV.

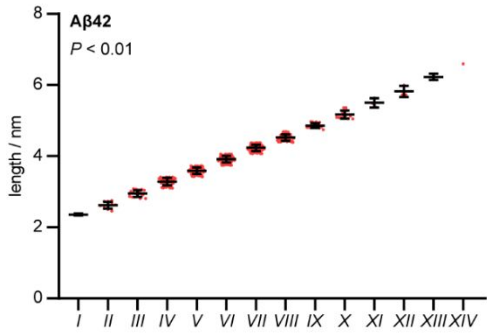

Fig. S20 Length distribution of the Aβ42 β-strands determined from the STM images in Fig. S19.

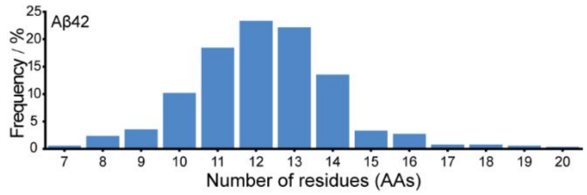

Fig. S21 Population distribution of Aβ42 β-strands in different amino acid residue number (N = 511).

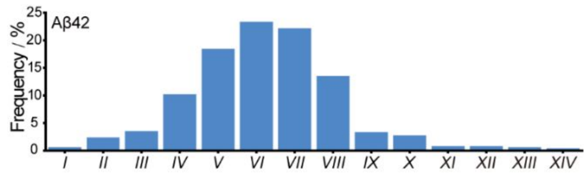

Fig. S22 Population distribution of Aβ42 conformational sub-states (N = 511).

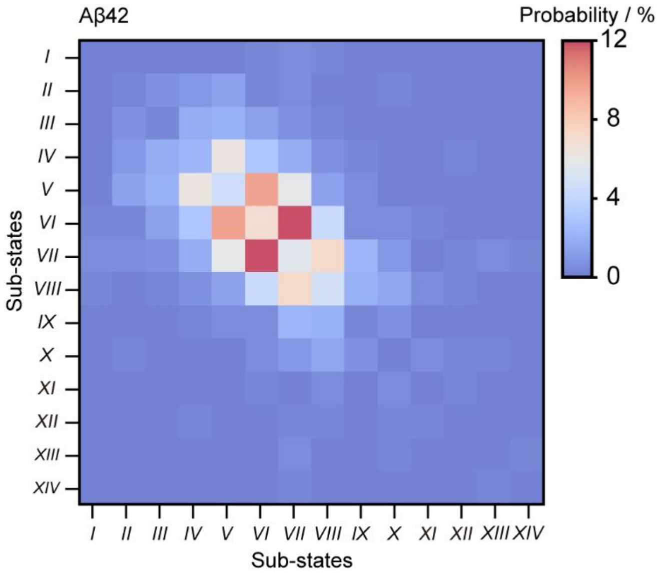

Fig. S23 Number distribution of different types of inter-conformation interactions identified from the STM experiments for Aβ42 (N = 460).

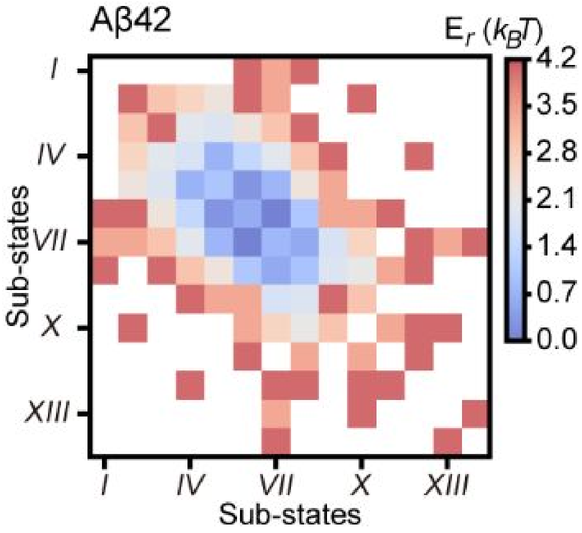

Fig. S24 Energy landscape of the inter-peptide interactions identified from the STM experiments for Aβ42 ($N = 460$).
